# Method of Depriving Quinine of Its Bitterness

**Published:** 1850-07

**Authors:** 


*Method of Depriving Quinine of its Bitterness.*—We find, in the April number of the American Journal of the Medical Sciences, a letter from Dr. R. H. Thomas, of Baltimore, addressed to Dr. Isaac Hays, on a method of depriving quinine of its bitterness. The "extemporaneous prescription," Dr. Thomas is in the habit of ordering, for a child two years old, is:

℞. Quinine sulph., grs. x.;
Acid. tannici, grs. ij.;
Aqua, ℥vj.;
Syrup. aurant. ℥iij.;
M. Dose—a teaspoonful every hour or two.

Mr. Stewart, of Baltimore, says that ten grains of quinine may be deprived of its bitterness, in a great degree, by the addition of one grain and a half of tannic acid.

We wish some one would discover a means of depriving quinine of its strong silvery taste, the strength of which has greatly increased in the past twelve months.—It is a heavy tax on the poor at this time.

